# *‘I was eager to do anything I could to improve the situation’*: a qualitative study of patients’ experiences and views of prehabilitation for ovarian cancer surgery

**DOI:** 10.1186/s12905-025-03630-5

**Published:** 2025-03-15

**Authors:** Rhia Kaur Saggu, Clare Shaw, Cathy Hughes, Pernilla Lagergren, John Butler, Alison H. McGregor, Sadaf Ghaem-Maghami, Mary Wells

**Affiliations:** 1 Department of Nutrition and Dietetics, Imperial College Healthcare NHS Trust, Charing Cross Hospital, Fulham Palace Road, London, UK; 2 Royal Marsden and Institute of Cancer Research Biomedical Research Centre, London and Sutton, London, UK; 3 Women’s Health, Imperial College London, London, UK; 4 Division of Women’s, Children’s and Clinical Support, Imperial College Healthcare NHS Trust, London, UK; 5 Department of Surgery and Cancer, Imperial College London, London, UK; 6 Department of Molecular Medicine and Surgery, Karolinska Institutet, Stockholm, Sweden; 7 Gynaecological Unit, The Royal Marsden Hospital, Fulham Road, London, UK; 8 Musculoskeletal Lab, Department of Surgery & Cancer, Imperial College London, London, UK; 9 Department of Surgery and Cancer, Imperial College London, Hammersmith Campus, Du Cane Road, London, UK; 10 Nursing Directorate, Imperial College Healthcare NHS Trust, Charing Cross Hospital, Fulham Palace Road, London, UK; 11 Department of Surgery and Oncology, Imperial College London, London, UK

**Keywords:** Prehabilitation, Ovarian, Cancer, Rehabilitation, Debulking, Surgery, Qualitative

## Abstract

**Background:**

Prehabilitation has shown promise in improving post-operative outcomes for several solid tumour groups. However, prehabilitation programmes are not widely established. Patients with advanced ovarian cancer experience life changing debulking surgery and could benefit from prehabilitation. This study aims to explore the views, experiences, facilitators and barriers surrounding prehabilitation in a demographically diverse cohort of advanced ovarian cancer patients. This would help to inform an acceptable patient-centred working programme model for a diverse group of patients.

**Methods:**

Purposive, maximum variation sampling was used to recruit a diverse sample of women, due to undergo or following primary debulking surgery for advanced ovarian cancer, from two cancer centres in London. Semi-structured interviews were either conducted face to face or by telephone. All recordings were transcribed verbatim and analysed using thematic analysis.

**Results:**

Twenty-one participants were interviewed. Twelve were prehabilitation ‘naïve’ and nine had participated in the Marsden Integrated Lifestyle and Exercise programme (MILE). The age range was 46–76 years and 8/21 participants were of Black, Asian or Mixed heritage. Factors influencing engagement with prehabilitation can be categorised under four major emerging themes [1] Mindset [2] Actual preparation [3] Support system [4] Delivery of prehabilitation.

**Conclusion:**

Patients with ovarian cancer welcome the concept of prehabilitation, however a blanket approach is not suitable to meet the needs of a demographically diverse cohort. The components of prehabilitation must be tailored to individual needs, with attention to existing mindset and support systems, building on preparations that women are already making for surgery and offering flexible delivery options.

**Supplementary Information:**

The online version contains supplementary material available at 10.1186/s12905-025-03630-5.

## Background

Prehabilitation programmes offer the opportunity to improve patients’ physical and mental function, by minimising the deconditioning related to cancer and its treatment, between the time of diagnosis and treatment [1]. UK National guidance published by Macmillan Cancer Support, the Royal College of Anaesthetists and the National Institute for Health Research Cancer and Nutrition Collaboration in 2019 recommended that prehabilitation should be incorporated into routine cancer care, with the aims of empowering patients to maximise resilience to treatment and improve long-term health [2]. However, it is recognised that several barriers to implementation exist, and that further research is required to strengthen the evidence base for prehabilitation, including an in-depth understanding of patients’ experiences [3].

It is particularly important to understand which patients are likely to benefit from prehabilitation. Ovarian cancers, in particular those in advanced stages, are associated with increased mortality and morbidity, often due to late presentation [4]. Women frequently present with deconditioning related to ascites, pelvic pain, cachexia, and loss of appetite [5]. Despite these problems, and the life changing impacts of ovarian debulking surgery, prehabilitation programmes for this patient group are limited [6]. It is now recommended that multimodal prehabilitation, comprising of nutritional assessment and intervention, physical activity and psychological support should be a recognised component of the pre-operative management of patients with advanced ovarian cancers [7]. Whilst there is no evidence from completed randomised controlled trials for multimodal prehabilitation in advanced ovarian cancer, there is research in progress [6]. Current models of prehabilitation for this group vary, and of those that exist, few suggest that patient co-design or theoretical models have been used in their development [8].

Qualitative studies exploring the feelings, influencers and barriers of women with gynaecological cancers have been conducted in mainland Europe [9–12] and the USA [13], but none in the UK. Most studies into the use of prehabilitation have included mixed cohorts of colorectal and ovarian patients, with only one study focussing solely on patients with ovarian cancer [13]. This study was limited by including White women only, all of whom received neo-adjuvant chemotherapy.

The findings of existing studies are not necessarily transferable to other cultural, demographic or treatment contexts. International differences in ovarian cancer treatment pathways exist [14], and even within the UK, some centres are more likely to use neo-adjuvant chemotherapy and interval debulking surgery, with others favouring primary debulking surgery. The difference in the time available for prehabilitation prior to surgery, due to the inclusion of neo-adjuvant chemotherapy, has the potential to impact on a patient’s perceptions and experiences.

The aim of this qualitative study was to explore the views, experiences, facilitators, and barriers surrounding prehabilitation in a demographically diverse cohort of women with a suspected or confirmed diagnosis of ovarian cancer who are either naïve to prehabilitation or who had experience of prehabilitation prior to debulking surgery.

## Methods

The core research team comprised of an experienced qualitative researcher (MW) and a novice qualitative researcher (RKS) who were clinicians in nursing and dietetics, respectively, but were not working in the gynaecological oncology field, therefore had no recent experience of prehabilitation within this speciality. Additional support with preliminary analyses was provided by experienced mixed-methodology researchers and clinicians, several of whom were specialists in ovarian cancer. Six patient and public advisors from Ovacome and Ovarian Cancer Action charities contributed to the study design and supported the development of study materials.

### Context

The research team had previously conducted a scoping review of multimodal prehabilitation for gynaecological cancers, using a realist perspective to understand the related barriers and facilitators to engagement and delivery that should be considered when designing a prehabilitation intervention for this group of women [6]. The findings of this review helped to inform the topic guide used in the current study, however, they were not used ‘a priori’ in coding or thematic analysis. The research team do acknowledge though, that their own research experiences and awareness of the existing literature will have influenced their approach to the analysis.

### Ethics approval and consent to participate

The study was approved by the Health and Care Research Wales Research Ethics Committee and Health Research Authority (IRAS 304833, REC 21/NE/0231). Local permissions were in place for participant information sheets and consent forms to be provided to prospective participants by care teams at a large teaching hospital trust and a specialist cancer trust within the NHS in London, either in person or via email. All participants were permitted to provide written informed consent in person or verbal consent over the telephone.

### Sampling strategy and recruitment

Participants were recruited from two participating sites, using purposive, maximum variation sampling to achieve a cohort which was diverse in age, social deprivation (by postcode), ethnicity, occupational status and experience of prehabilitation. Both participating sites were major cancer centres within the National Health Service (NHS) in London. One site had no pre-established prehabilitation programme whilst the other site offered patients the opportunity to participate in a multimodal programme called the Marsden Integrated Lifestyle and Exercise (MILE) up to 3 months prior to their surgery. This programme offered patients physical activity guidance, psychological support, and nutritional advice through videos and worksheets to help them prepare for treatment. Patients were referred to specialists such as physiotherapists, dietitians, or psychological support services if additional tailored interventions were required beyond the standard programme. Referrals were based on individual needs, as identified by healthcare professionals, and appointment availability varied depending on caseloads and waiting times.

At each participating site, the gynaecological cancer Clinical Nurse Specialist (CNS) team screened the respective surgical lists to identify potential participants using the eligibility criteria. Lists of patients on the MILE programme were re-screened to ensure that we included women who had participated in at least one specialist prehabilitation session and could therefore discuss their experiences. Women with suspected or confirmed stage 3 or 4 ovarian cancer who were due to undergo or had already undergone primary or interval debulking surgery as part of their treatment at either of the two participating NHS Trusts were included in the study. Exclusion criteria included those with recurrent disease undergoing secondary debulking surgery, or oncological treatment only and participants who lacked mental capacity to consent to participation; were non-English speaking and were below the age of 18 years.

All potential participants were then contacted by their CNS team, either via email, telephone or in person, to invite them to the study and request permission to be contacted by the research fellow (RKS). In the case of non-response, an individual reminder was sent. All prospective participants who agreed, received a copy of the participant information sheet and consent form followed by a phone call from the research fellow to answer any further questions and schedule a date for the interview. All consent was taken on the day of the interview. Written consent on paper was given for all participants interviewed in person and verbal consent was given over the telephone for those interviewed remotely. None of the interviewees were known to the primary researcher (RKS) and main supervisor (MW) in either clinical or research capacities.

Recruitment continued until data saturation was apparent. This meant that RKS and MW were in agreement that new topics were not being raised during interview, new codes were not being identified during initial coding and that the collected data was sufficient to adequately address our research question [15]. All participants were reimbursed with a shopping voucher worth 25 British pounds, for their participation.

### Study design

Semi-structured interviews were led by the first author using a topic guide that was developed in collaboration with the patient and public advisors (supplementary material). Questions encouraged interviewees to reflect on the time between diagnosis and surgery, including how they felt and their priorities during this time; their preparation for surgery, and their thoughts, experiences and views of prehabilitation as well as their goals for recovery.

To encourage participation, interviews were offered remotely via phone or face to face on the surgical ward, as per interviewee preference. All participants consented to the audio recording of their interviews, which were anonymised and sent for transcription to an external company (WayWithWords).

Data were analysed using Thematic Analysis [16]. Initial inductive coding of the transcripts was completed by the primary researcher and findings were discussed and developed with support from a second and third researcher; following which, an initial coding tree was developed using NVivo software. Codes were discussed and initial themes and sub-themes were created and then reviewed by the research team. The Standards for Reporting Qualitative Research (SRQR) checklist [13] was used to guide reporting.

## Results

**Fig. 1:**
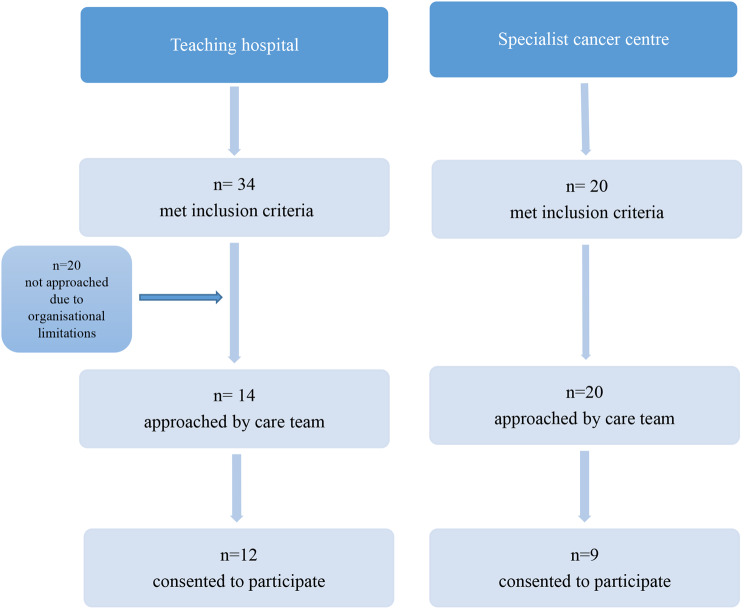
Flow diagram illustrating the process of participant recruitment at each participating site

Twenty one patients from the specialist cancer centre and 23 patients from the teaching hospital within the NHS in London, met the inclusion criteria and were invited to take part in the study (Fig. 1). Of these, 9 from the specialist centre and 12 from the teaching hospital (total 21 participants) consented to be interviewed. Interviews took place between March and July 2022; 8 in person on the surgical ward and 13 over the telephone. Interview duration ranged from 24 to 83 min (Median 54 min). Interviewee demographics are provided in Table 1.

**Table 1:** Participant characteristics based on their experience with prehabilitation and surgery as well as demographics

Participant	Prehabilitation	Pre/post-operative	IMD* Decile	Age	Ethnicity	Occupational Status
1	Naïve	POST	4	57	White-British	Student
2	Naïve	POST	7	56	Asian-Indian	Sick Leave
3	Naïve	PRE	10	72	White- British	Retired
4	Naïve	POST	6	65	White- British	Sick Leave
5	Naïve	PRE	3	51	Black-Caribbean	Sick Leave
6	Naïve	PRE	9	76	White- British	Retired
7	Naïve	POST	3	55	Asian-Pakistani	Retired
8	Naïve	POST	3	46	White- British	Sick Leave
9	Naïve	PRE	6	76	White-British	Retired
10	Naïve	PRE	2	52	Black-African	Voluntary work
11	Naïve	PRE	7	45	Asian- Other	Sick Leave
12	Experienced	POST	9	72	White-British	Unemployed
13	Naïve	PRE	6	58	Asian- Indian	Sick Leave
14	Experienced	POST	5	55	Mixed	Self-employed
15	Experienced	POST	10	74	White- British	Retired
16	Experienced	POST	9	48	White-British	Unemployed
17	Experienced	POST	8	79	White-British	Retired
18	Experienced	PRE	3	56	Asian-Other	Sick Leave
19	Experienced	PRE	10	66	White- British	Retired
20	Experienced	POST	4	68	White-British	Retired
21	Experienced	POST	4	59	White-Other	Sick leave

Of the participants who had experience of prehabilitation, all provided insight into the impact it had on their pre-operative preparation. However, the data do not point to specific benefits of individual components of prehabilitation but instead illustrate the importance of the context in which prehabilitation is offered and delivered.

**Fig. 2:**
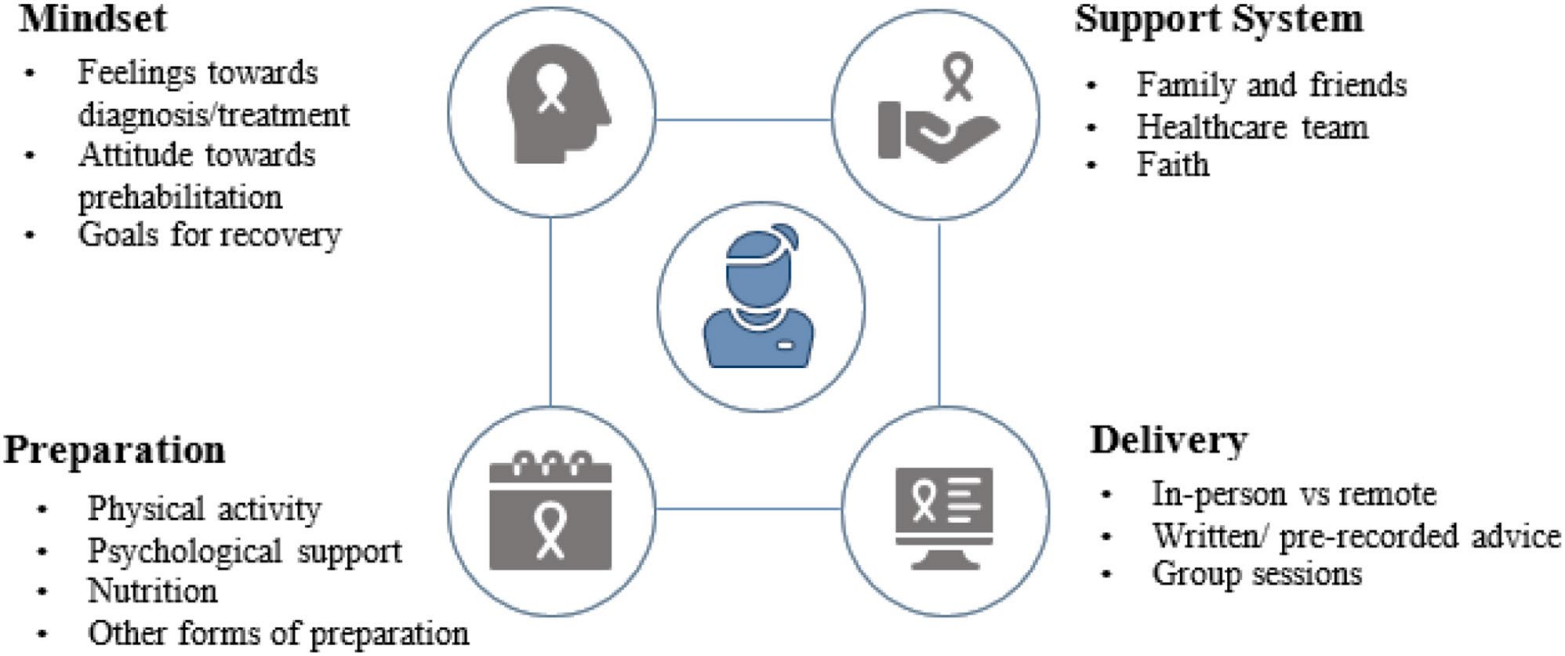
Overarching themes underpinning the views, experiences and influencers surrounding engagement with prehabilitation as means to achieving personalised

This context encompasses the views, experiences, and influencers around engagement with prehabilitation, which can be summarised under the following major themes [1] Mindset; [2] Actual preparation; [3] Support system; and [4] Delivery of prehabilitation (Fig. 2). Participant quotes, classified by the participants’ experience of prehabilitation (naïve or experienced) are provided in the text and Table 2.

**Table 2:** Participant quotes relating to the respective sub-themes

	Feelings towards diagnosis and treatment
Participant 11, naïve.	*‘Some of it was shocking to find out. And after that, again, it took me, I would say, around a week or so to accept what has happened. And then one day I remember I was saying, why me? And then I suddenly realised, why not me then?’*
Participant 13, naïve	*‘Yes, I’m just preparing my bag and myself. I was just thinking whatever has happened, it has to happen for the best. And I have to trust in the surgeon, and that’s it.’*
Participant 15, experienced	*‘So it was just process, that’s what was going to happen, this has got to happen. And because there was so much more going on, it took the emphasis out of the sting in the tail, if you know what I mean. But yes, I just carried on. Just got to do what you’ve got to do. Yes, we all got a little bit upset and worried. But I was told at the time it was curable, so that was what I kept in my mind. And that’s what got me forwards.’*
Participant 16, experienced	‘*Well, I think to stay positive, it’s a mindset you need to be in, I think. And, fortunately, that wasn’t very difficult for me. But the attitude of mind has got to be the right one, I think, and full knowledge of what to expect, so that you’re not given any surprises along the way, so to speak. Better to know everything than only bits and pieces.’*
	Attitudes towards prehabilitation
Participant 3, naïve.	*‘I think I would be responsive, because I always want to do the right thing. I would consider it a priority, really. It’s necessary to keep yourself in the best possible condition you can be, to face whatever’s coming and then get back to doing all those other things, like grandchildren or whatever. If you’re only halfway there, the prognosis can’t be as good if you don’t go into it the best you can be’*
Participant 10, naïve	*‘I didn’t receive any exercise advice or food or… I didn’t get anything. That would be good, I think’.*
Participant 12, experienced	*‘I was very eager to do anything I could to improve the situation. Absolutely if they’d told me whatever I would have done because anything that helps has to be to my benefit, doesn’t it, at the end of the day. So yes I was very willing to make the changes.’*
Participant 18, experienced	*‘It was so useful, and you feel supported as well. And you feel that there’s people that is concerned about you, there’s people out there who think that you need this help. I think this Mile programme is one of the best, and will recommend this to anyone who gets cancer because you feel. I didn’t know there is lot of help like that. When they talked for me they are got this programme there’s a physio, the dietitian, I’ve got this letter about the psycho, I was like, oh yes there’s good things in this world’.*
	Goals for recovery
Participant 4, naïve	*‘Tennis, because I’m an avid tennis player, and that is my goal, is to get back on court.’*
Participant 5, naïve	*‘When I think back now, autopilot, literally autopilot. I still feel like I’m running on autopilot. It hasn’t really caught up with me yet. I think maybe after everything’s done, I might need just time out before I go back to work just to catch up with myself and to deal with what I’m going through, what I have been through, etc. But right now, I’m literally just going on autopilot. That’s what I feel I’m doing. So, I don’t know.’*
Participant 15, experienced	*‘Just being myself. Just being back to me again without having to think about it. To be fair and honest, my recovery isn’t how I imagined it. But what pushed me on was that I was going to get back to how I was, just carry on with life just as before diagnosis, before I was poorly. I’m quite an active person. I like being in and out and busy all the time. But it hasn’t been the way that I thought it would be. But that’s how I envisioned it, that I would just be back to normal, back to myself.’*
Participant 19, experienced	*‘I’m hoping I’m not going to be one of these people, oh what if it comes back? I don’t really want to think about that, I just really want to have a bit of normality back, and for my family as well’.*
Participant 21, experienced	*‘Early 60s, as I am now, I’m thinking maybe you can go back to Spain and put your own business. I know that I don’t want to go back to what I was doing before. Even if I have to, because obviously, like I say, I don’t know the treatment, how it’s going to be from here, from now to the future. And I don’t know exactly when I can go or not go or do or not do, but I’m going to try to do something different.’*
	Physical Activity
Participant 17, experienced	*‘[Physiotherapist] wanted to make sure that I was fit enough for surgery so I had to do some from a sitting state to stand up and down for the period of a minute, and they did that twice. And when they discovered the second time I was doing really well they said they didn’t think that I needed the intervention anymore which I probably don’t, I have been quite fit in my life’.*
Participant 20, experienced	*‘[Physiotherapist] was never too busy to talk to me to see what my restrictions were, to see what I could do. He was very able to modify the programme he was thinking of to include some of the things I have issues with, and engaged a lot with what I wanted’.*
Participant 15, experienced	*‘Rather than someone, talk about your feelings, and understanding how you feel if you’re upset… you feel better in yourself just by doing the extra class, or going for that extra walk, or whatever.’*
Participant 10, naïve	*‘I used to walk more than two hours a day but now I couldn’t even walk… because I have to go back to the toilet because I couldn’t control my bladder because of the fluid in my stomach. And I get tired quickly.’*
	Nutrition
Participant 8, naïve	*‘I can’t remember them specifically saying, reduce my alcohol intake, but I just thought my body’s going to be really bombarded with drugs and is going to need to be in the best possible position to heal, so I felt I ought.’*
Participant 14, experienced	*‘The other thing that worked hand in hand was I had a conversation with the dietitian, she would call me every maybe three weeks or so. We would talk about meals, we would talk about healthy eating, we’d talk about the ratio of vegetables to other things, and we’d just have a proper full discussion. And she gave me a tip when I first spoke with her to keep a food journal, just so that when we spoke we could look at it and see what improvements could be made.’*
Participant 17, experienced	*‘They invited me to have an interview with a nutritionist, but once again I had to wait for eight weeks. There didn’t seem to be any point quite frankly. Because actually I think my diet’s pretty good, and I’d had this interaction with the surgeon who told me very clearly I needed to put on some weight. So actually I just followed his advice and said to the nutrition lot thank you very much’*
Participant 12, experienced	*‘For me no. I know loads of people who’d jump at it. But for me no, it wouldn’t, I don’t think that would work for me. I think it would make me think things that I don’t want to think. It makes you more conscious of things.’*
Participant 14, experienced	*‘ I’m still actually having psychotherapy right now, and I think it’s hugely beneficial, because what it did was it brought it all into context, what I’d been through, what happened pre-surgery, and what happened after surgery. So, although I was supposed to start the psychotherapy before surgery, I personally thought it worked really well for me having it after surgery.’*
Participant 17, experienced	*‘Maggie’s are absolutely amazing. I just felt so much at home from the minute I got there. They arranged for me to see their psychotherapist. You see once again it was face to face which was wonderful. And he listened to my requirement and my story and he said, I think we can work with you, I’d like to invite you to a series of appointments with me’*
Participant 21, experienced	*‘Well, there was a space, a Maggie’s centre, where you can actually pop in and talk to someone there. But I was a couple of times. The psychologist wasn’t available at that point because you don’t make appointment. You just go and talk to her. I spoke to someone there, but I don’t know, I thought that I need more than that. I don’t need just a quick chat. At that point, it was hard.’*
Participant 19, experienced	*‘Sometimes you can talk to people you don’t know very well a little bit better than… your family. It’s just that you don’t want to worry them, or you don’t want their whole life to be consumed by the cancer.’*
	Psychological support
Participant 12, naïve	*‘For me no. I can still that it would be very useful for loads of people, I know loads of people who’d jump at it. But for me no, it wouldn’t, I don’t think that would work for me. I think it would make me think things that I don’t want to think. It makes you more conscious of things. Yes, so not for me but I can see that it would be very useful for a lot of people.’*
Participant 14, experienced	*‘So I think the psychotherapy, depending I guess on the individual, but certainly for me, it just was the thing that rounded it all up and brought it all home to make me this fulfilled person ready to live my best life again.’*
Participant 15, experienced	*‘I was offered that that I could always go and speak to someone, they could arrange for me to go and speak to someone. But honestly, I have the most supportive family that listen completely that I didn’t feel that I needed that.’*
Participant 16, experienced	*‘No, I didn’t. I certainly was aware of it, and right from Day 1, I was aware of it, if I’d wanted to. But I didn’t, to be honest, feel the need for it, personally. This is just me, and I didn’t take it up at all.’*
Participant 17, experienced	*‘I always come away with something new, and it’s either a new attitude, it’s mostly a new attitude. It’s been about problem-solving. It’s been about thinking through things in a way that I haven’t done before. It’s always about considering not just my own point of view but other people’s. I don’t complain a lot, but if I get on to the edge of complaint he will always open my mind as to how it is for the person on the other side and why this might be happening.’*
	‘Life’ preparation
Participant 12, experienced	*‘I just thought I needed to sort myself out legally… Getting my will straight, that sort of thing. It’s one of those things that sort of hangs over you and you never quite get round to it but it spurred me on which was probably a good thing, actually.*
Participant 1, naïve	*‘I’ve got my kids at home this week, and also my dog is very poorly, so actually there were other things in my head that were more prominent for me than having the surgery.’*
Participant 3, naïve	*‘I have written my husband and my two children notes, and I’ve said where they are, in case something happens tomorrow.’*
	Friends and family
Participant 16, experienced	*‘Well, I don’t have children. I am married. My husband was brilliant. He was probably in more of a state of shock than I was about it all. But he was and is fully supportive and he’s been 100% behind me, obviously, in it and helping with everything, really, which makes, obviously, a difference. Because if you’re doing this on your own, it could be quite hard.’*
Participant 14, experienced	*‘He’d [husband] say why don’t you put your gym clothes ready the night before, so in the morning you can just hop into them…And then for diet, because he did all the cooking and the food shopping for us, he also was in the conversation, the first one. Not all of them, just the first one with the dietician, where we talked about foods and food groups, and so on, and so he then knew.’*
Participant 2, naïve	*‘And the main thing is my family supported lots. They are normal. Nothing has changed in my house. Everyone’s routine life is the same. They never give me any sympathy. They give me strength.’*
Participant 21, experienced	*‘He loves me a lot, but the diagnosis for him, the impact was worse. So, he mainly just takes care of me physically. The food, the things. But mentally, I think, he needed more. I stopped fighting him for help. Poor thing. But when I was at home, he started to feel better about all these things.’*
	Healthcare professionals
Participant 17, experienced	*‘Well that was probably the most useful thing I had because first of all the anaesthetist herself was very approachable, she was very professional. She didn’t hurry me, she gave me the feeling that I’d got all day to talk to her, I can’t think how she did that because she must be terribly busy. And she was very, very down-to-earth.’*
Participant 20, experienced	*‘I don’t have a good rapport with the gynae nurse practitioners because we don’t have a relationship, they answer questions and that’s probably it. They were very honest that there were these shortages, they didn’t not tell me that this was going to be problematic, but I don’t necessarily know that. The health service is in crisis but we can only do what we can’.*
	Faith
Participant 20, experienced	*‘I had gained an ability to centre myself and calm myself. I have extremely good control normally as my faith is, and they are all the appropriate skills that you need. So I practised prayed them much more from Asanas to Samadhi and just acceptance and keeping the positive feel of it. And I’m not saying that it was particularly easy, but I have not been crippled by this diagnosis, it’s been a project to work through.’*
Participant 7, naïve	*‘I do listen to religious music to stay calm, especially when I think I’m getting a little anxious. So, it helps me to relax. And I guess, knowing and then telling myself. I’m only a drop in the ocean… I guess it just makes me feel good to know that’*
Participant 2, naïve	*‘I’m telling everyone, go to positive and believe in God. If you are strongly believing in God, faith and trust, they’re never going to disappoint you.’*
	In person vs. remote
Participant 15, experienced	*‘The time you’ve been to the hospital for chemotherapy all day long, and you go for your blood test, and you go for a scan, another appointment on top, from my point of view, is just too much. So it’s quite nice to be able to still have that reach to speak to somebody. But it’s quite nice to be doing it at home rather than in the hospital environment.’*
Participant 19, experienced	*‘I think they need to see you as well to see how you’re coping maybe, because we can all put on a front over a phone and say, oh yes I’m fine. I think if they see you face to face they can judge a little bit better maybe.’*
	Written or pre-recorded advice
Participant 8, naïve	*‘You have physio, dietician and psychologist or whatever, but maybe like a generic pre-recorded session, with then opportunities for people to do like a virtual drop in to ask any questions after they’ve seen the videos. Or an email address, like the Macmillan nurses have, where it gets checked regularly, and you can respond to any queries that people have.’*
Participant 21, experienced	*‘So, like I said, having it online is really helpful, especially if it’s live, because if you have to put a pre-recorded video or something, it doesn’t motivate you as much as to know that you’ve got a time to connect to do the class. And there are people there live with you. That helped a lot as well.’*
	Group sessions
Participant 14, experienced	*‘For the exercise part, and we are all working to the same goal, i.e. to get as fit as we can and as healthy as we can before surgery… But for me personally I think it would have been helpful just for that, because I wouldn’t have wanted to compare cancers, or stages, because that would send me into a downward spiral.’*
Participant 12, experienced	*‘No, no I don’t think so, I don’t think I would do that. Because then it gets sort of a therapy session doesn’t it, really. And I think in some ways I’m better off not knowing what happened to another person’*
Participant 11, naïve	*‘It’s always good to talk to people. Maybe things that the other person is doing that you’re not doing, and its benefitting maybe it’ll benefit you as well. And yes, the other way of networking as well. It’s always good to talk to people who’s been through that as well.’*

### Mindset

This theme describes the mindset of patients at various stages of their cancer journey and the impact this may have on engagement with a prehabilitation programme. This theme is explained through 3 subthemes: [1] Feelings towards diagnosis and treatment [2] Attitudes towards and perceived need for prehabilitation and [3] Goals for recovery.

### Feelings towards diagnosis and treatment

Amongst the interviewees, more than half the interviewees expressed a conscious choice to stay positive as a key coping mechanism in response to their cancer diagnosis.

*‘Well I’m quite a strong person actually and I did cope and I sort of mentally prepared myself really. Once I knew I was going to have surgery I said okay fine, I’ve got to change my mind-set and be positive.’– P12, experienced*.

Whilst the diagnosis was considered a shocking experience, participants took solace in knowing that surgery was an option for them.

*‘…at the back of my mind I’m thinking I’m cured already you know, because I’m a suitable case for treatment.’- P6, naïve*.

In turn, being offered treatment became a motivating factor for patients to optimise their fitness to be a successful candidate for surgery.

*‘I think the fear just made me want to eat healthier, be healthier…My biggest fear was when they said to me that I wouldn’t be able to have the surgery if it hadn’t shrunk enough.’– P15, experienced.*

### Attitudes towards and perceived need for prehabilitation

The majority of interviewees described already being engaged in physical, nutritional and/or mental preparation for surgery, irrespective of whether they referred to it as ‘prehabilitation’ or not. Women who were prehabilitation naïve were generally positive about the idea of a formal prehabilitation programme and would consider taking part if it was offered to them. Those enrolled on the MILE, reflected on their diagnosis being a teachable moment for them, and felt that prehabilitation guided them in improving their lifestyles.

‘*If I was given the opportunity, yes, I would have straightaway taken. But I was not given any advice on that.’- P11, naïve.*

*‘… it’s such a shame it took this for me to do it, to actually get fitter, lose a bit of weight, eat properly. I should have done it years ago, and I’m quite cross with myself that it took something like this to make me open my eyes and be a bit healthier.’–P19, experienced.*

However, enrolled participants still spoke about the importance of having the prehabilitation pathway explained to them to fully understand its role in preparing them for surgery.

*‘I was a bit confused about it really because I don’t think it was very well explained to me. Well I did understand that they were trying to establish whether I needed further input before surgery, I think that’s how I would put it, and obviously they decided I didn’t.’–P17, experienced.*

### Goals for recovery

Goals for recovery revolved around outstanding tasks, spending time with friends and family and travelling. The majority, irrespective of age and occupational status, spoke about the concept of ‘returning to normality’ as their primary goal for recovery, which dovetailed into their post-operative fitness goals.

*‘It’s about coming back to humanity as quickly as possible. And obviously first of all it’s being able to manoeuvre around.’- P1, naïve.*

Alternatively, some interviewees rejected the notion of looking towards normality as an end goal and that choosing to take one step at a time was considered more realistic in the context of uncertainty and reappraisal of life.

‘*But I think I don’t want to come back to the life as it was. That’s why maybe I don’t think further ahead. It’s just I’m thinking the day as it comes and I’m figuring out what I’m going to do next. Normality for me is not going to be enough…You realise that life is short.’–P21, experienced.*

### Actual Preparation

All described ways in which they had prepared for surgery. The theme of ‘Actual preparation’ is further explained within each component of multimodal prehabilitation [1] Physical activity [2] Nutrition [3] Psychological support as well as [4] ‘Life’ preparation.

### Physical activity

Of those who spoke about their experience of preparing for surgery, either staying, or becoming, more physically active was more widely described than the other components of prehabilitation, whether or not they were formally enrolled in a prehabilitation programme.

For those who were prehabilitation naïve, walking, resistance exercises and domestic tasks were the main forms of physical activity. Any guidance around fitness was usually provided by the CNS on initial consultation:

*‘I was fully aware that I needed to keep myself active, but for me being active was cleaning the windows, or doing the housework, or pottering around in the garden, that for me was my way of exercise.’- P9, naïve.*

Interviewees from the MILE programme described themselves as physically active at baseline. For some, this meant that physiotherapy support was not required, either because they were not considered at risk at the point of screening or because they declined additional support through the programme.

Those who did avail physiotherapy found that it both improved their knowledge surrounding fitness and encouraged movement. For those with health restrictions, guidance was tailored to fit their ability. Additionally, exercise was felt to have a positive impact on mental health during treatment.

*‘So it did have a positive impact, my physical fitness 100% improved… I think this helped me to realise the importance of movement and the importance of having a strong body, regardless of if I felt really bad during chemo…’–P14, experienced*.

The biggest barriers to engagement with physical activity were tumour related comorbidities, in particular ascites and fatigue.

### Nutrition

Most interviewees considered their diets to be ‘healthy and balanced’, often consisting of a high intake of vegetables and a limited intake of processed foods and meat. In fact, some interviewees spoke about turning vegetarian and/or making self-directed choices about their diet following their diagnosis, in an attempt to become ‘healthier’.

*‘I eat pretty healthily anyway but I gave up meat… I suppose I just thought about it more really. And just became more conscious of it and making fresh foods.’–P12, experienced.*

There was a mixed response to the dietetic intervention provided through the prehabilitation programme. On one hand, interviewees benefited from tailored advice provided by the dietitians, especially with regards to colostomy management, micronutrient intake and food portions. On the other hand, patients who felt confident managing their own diet were happy to continue without professional advice, whilst others could not recall any impact that interacting with the dietitian had on their preparation for surgery.

### Psychological support

Psychological preparation involved practising relaxation, listening to audiotapes, and meditating. Preparing mentally for surgery by seeking formal psychological counselling was the most controversial component of prehabilitation. Those who were not offered this intervention, or declined psychotherapy within the MILE, displayed uncertainty around its benefits.

*‘I can’t think, really, what a professional would say, other than keep calm and just carry on.’– P3, naïve.*

On the other hand, those who did receive psychotherapy through the MILE programme or at the Maggie’s Centre^1^ found it useful for coming to terms with their current situation.

Unfortunately, some interviewees reflected on their need to receive psychological support prior to surgery, but in view of extensive waiting times, were unable to speak to a professional in time.

*‘I asked for some help in hospital, and they said they would refer me but nothing ever happened, and I think that was probably my biggest disappointment, I could have done with it then. I understand that the services are very pressed but for something like that you don’t need to wait.’- P17, experienced.*

Some interviewees suggested that cultural taboos around gynaecological cancers and reducing familial burden were factors which encouraged engagement with psychological support.

*‘I come from an ethnic minority society, where in African households you don’t speak openly, especially gynaecological cancers…. So, already I’m muffled in a way where I can’t express my inner concerns.’- P14, experienced.*

### Life Preparation

For many patients, preparation for surgery extended beyond the accepted components of multimodal prehabilitation. Interviewees spoke of domestic and legal tasks e.g. will writing, funeral planning and financial tasks taking priority during the time before surgery. In certain situations, women prioritised their family’s needs and wellbeing before their own, ensuring everything was organised prior to their departure for surgery. Interestingly, there was little mention of self-care and self-prioritisation during this time.

*‘I’m not a control freak at all, by any stretch, but I have to in my mind know that I’ve left the house as good as it can be, that my husband hasn’t got to do anything.’–P9, naïve.*

### Support system

Having a reliable support system was found to be an influencing factor in perceived engagement with prehabilitation, as well as providing social and moral support during a difficult time. Support systems were described as [1] Friends and family [2] Healthcare team and [3] Faith.

### Friends and family

Friends and family, particularly the latter, were highly regarded as a support system in women’s preparation for surgery. Interviewees spoke extensively about the domestic role their partners and children took on, to relieve their workload burden, be it cooking, cleaning or grocery shopping. Family and friends were also considered key motivators to participating in prehabilitation and provided physical and moral support in the lead up to surgery.

*‘My husband morally support me so well. I feel supported by him the way he talked to me, the way like throughout up to now when I go to do my chemo he will take me there and bring me back. When I come home he will support me there to do the house chores, cooking when I can’t. So I would say he is my right hand.’– P18, experienced.*

On the other hand, interviewees shared deep concern for their families; worried that their treatment and prognosis could adversely impact those around them. Women spoke of the impact their diagnosis had on the mental health of their male partners, specifically, and the need to overcome their cancer for the sake of their families, more than themselves.

*‘I have to be fine for my kids and my husband. I know I have to fight for myself but, at the same time, I’m thinking my kids.’– P10, naïve.*

### Healthcare team

The relationship they fostered with their respective care teams significantly influenced how well supported interviewees felt during their time between diagnosis and surgery. For the patients who reflected positively on their interactions with healthcare professionals, being given enough time to talk at length and discuss their concerns in the context of a humane conversation, was considered most valuable to them.

*‘We had a very long conversation about swimwear in one of the consultations. It was just hilarious. It made quite a nice change. he also then also assured me that sex would still feel the same after I’d healed, which was reassuring.’– P8, naïve.*

Alternatively, patients who did not receive the time they required from their care team due to resource constraints, felt less supported during their treatment journey.

*‘I do understand that they have other patients too, it’s not just me, and that’s what I keep trying to tell myself. So accessibility for me would be a big thing, because when I want to know something, I want to know it.’– P6, naïve.*

### Faith

Faith in the context of religion and doctrine teaching, across all ethnic backgrounds, was also considered an important support system to provide patients with perspective, motivate them through their cancer journey and reach acceptance of their diagnosis through divine communication.

*‘Because I was normally fit. When I was told, you’ve got cancer, I thought never happen to me and the only thing makes me strong, my faith, believe in God. So, I think for me that’s my number one, and praying and reading God’s words.’– P10, naïve.*

### Delivery of prehabilitation

A major factor surrounding engagement (or potential engagement) with prehabilitation was the way in which the service was or should be delivered. Interviewees held opinions on the following provisions [1] In-person vs. remote [2] Written or pre-recorded advice and [3] Group sessions.

### In-person vs. remote

Discussion largely focussed on the dilemma of whether prehabilitation should be delivered remotely or in person, and the related consequences. For patients who lived further from their treatment centre, or, who suffered with adverse side effects, a remote programme was considered more convenient and practical to attend. Some patients receiving neo-adjuvant chemotherapy at the specialist cancer centre reflected on the amount of time they spent in hospital attending appointments, so therefore, welcomed the opportunity to engage with the MILE programme at home. Furthermore, they encouraged the transition to the virtual world which has been accelerated by the covid-19 pandemic.

*‘I think we need to respect that there’s a pandemic going on. So I think if it’s not necessary for you to come in, you don’t have to. I think virtual works.’– P11, naïve.*

Some interviewees felt passionately about receiving their care face to face and in person to get the most out of their consultation with their healthcare professional. Concerns around accessing virtual platforms also highlighted an unintended consequence of tele-health.

*‘The physio I do believe it’s been difficult during COVID because everything is on the phone and you don’t have so much face to face. I’m a person who benefits hugely from face-to-face contact…I do online stuff, but no, by the time you reach 78 it’s not second nature. And so I’m used to the written word, and the spoken word.’- P17, experienced.*

### Written or pre-recorded advice

Interviewees expressed mixed views in response to receiving written advice or being signposted to pre-recorded material. All interviewees had received written information in the form of booklets and leaflets over the course of their treatment. Some patients valued this form of advice as they were able to share it with loved ones, refer back to advice in their own time and ask for clarification from others if English was not their first language. However, patients also recognised that written advice alone does not compete with the benefits of speaking to healthcare professionals.

*‘When you read through a tonne of text a lot of it is not going to be particularly relevant to you. When you have a face-to-face, it gives you the opportunity to ask something that you want to know, particularly.’– P1, naïve.*

In line with the views on remote delivery, pre-recorded advice was considered convenient, allowing people to access information in their own time and in the right frame of mind. However, this was disputed by those who felt that pre-recorded information removes the ‘personal touch’ of meeting people in person, and the motivational element of group sessions.

### Group sessions

Attending group sessions for the individual components of prehabilitation was discussed in detail and interviewees expressed mixed opinions on this method of delivery. Attending a group session, either in person or virtually, for the purpose of exercise, appeared to be well accepted by the interviewees as they felt that sharing a common fitness goal was motivating for participants. However, there were conflicting views about group therapy or general networking opportunities with other ovarian cancer patients. Interviewees suggested that instances in which cancer journeys are compared could provoke negativity whilst others felt it could be a source of knowledge.

## Discussion

This study set out to identify themes underlying the views, experiences and influencers surrounding engagement with prehabilitation in a diverse cohort of women who were either naïve to a formal prehabilitation programme, or enrolled in the MILE. Qualitative research such as this current study can be used to inform the co-design of complex interventions so that they are more relevant, acceptable and patient-centred, addressing the barriers and challenges that patients are likely to face [17]. This is consistent with the Medical Research Council [18] and O’Cathain et al.’s [19] guidance for developing and evaluating complex interventions.

Overall, participants displayed great positivity towards increasing their ability to withstand surgery, optimise recovery and improve the situation for their family. This in turn, meant women felt strongly about prioritising their physical wellbeing, either through self-determined choices or by engaging with a prehabilitation programme. However, interviewees expressed mixed opinions on services which could have unintended consequences for their mental wellbeing and displayed diverse attitudes towards psychological interventions, support systems and the way in which prehabilitation is delivered. This study confirms what we know about the benefits of remote delivery and also the view that preparation for surgery extends to legal and financial obligations [9–11]. However, our study has provided further insights into the varied feelings women have towards psychological coping strategies, their concern for their spouses and children and the role of faith as a pillar of support. De-prioritising prehabilitation in the context of limited time has been suggested elsewhere [6], but a lack of time was not described as a barrier to engaging with prehabilitation in this study; possibly because all interviewees who were in employment were assigned sick leave from work to manage their treatment.

Our study delves deeper into the factors which need to be addressed by the health care system for prehabilitation programmes to be successful. Importantly, women took the initiative to prepare their body and mind in ways which were acceptable to them. Some did not perceive the need for formal prehabilitation, either because they thought they were already doing what they could to prepare, or because their needs were different to those covered by the three pillars of multimodal prehabilitation. Other studies have also found that women engage in their own forms of preparation for treatment, including tasks which fit in to their everyday lives such as meal preparation, laundry and gardening [10, 11]. In these studies, women were ready to accept prehabilitation as being beneficial for health and wellbeing, but spending time with loved ones, funeral planning and finances were considered by some as equally important. Building on the different motivators which patients express e.g. family, faith, healthcare professionals, is likely to be instrumental in improving engagement with more formal prehabilitation, however, attention must be paid to the potential to overburden patients with responsibility for their own health at a particularly vulnerable time [3]. It is also important that clinicians provide meaningful information about the benefits of prehabilitation. Some participants in this study felt that this had not been explained well enough to them. A more personalised approach, linking individual goals to the pillars of prehabilitation is needed [20]. Moreover, attention to the relevance of prehabilitation within the individual’s social context is important for engagement [21]. Our findings support using the theory of self-determination [22, 23] to improve the chances of success when designing prehabilitation. This theory combines attention to autonomy, relatedness and competence, all of which are embodied in our findings.

However, utilising patient motivators to achieve personalised care requires understanding of the patient’s individual needs, beliefs and goals, possibly through initial screening questionnaires, assessments or consultations. No studies to date in ovarian cancer have looked at the use of a person-centred approach to prehabilitation. Future prehabilitation programmes are likely to be more effective if they consider individuals’ beliefs and needs, and use behavioural theory to underpin such an approach [24]. However, the economic implications of routine prehabilitation need to be evaluated, given the anticipated demands on healthcare professionals’ time. Our findings emphasise the importance of having responsive and timely clinical support and the value of face to face interactions with the healthcare team; these are important considerations in the light of workforce and financial pressures facing global healthcare systems, as well as the trend towards greater digital healthcare provision.

The strengths of this study are that we recruited an ethnically diverse sample of women with ovarian cancer and were able to interview both pre- and post-operative patients who had no experience of prehabilitation as well as those who had participated in a programme. Although we cannot be sure that our findings are generalisable to non-surgical patients with ovarian cancer, our themes were meaningful across our diverse sample, suggesting that they would be relevant to any prehabilitation programme designed for a surgical group. A limitation is that we cannot be sure that the prehabilitation ‘naïve’ patients would hold the same views of prehabilitation if they had actually been offered it or that patients would have reported similar experiences and views had they been interviewed at a different time in the pathway. Additionally, the exclusion of non-English speaking patients is a recognised challenge in obtaining representative views and experiences in this patient cohort. Unfortunately, demographic data on those who declined to take part was not consistently recorded, therefore we cannot comment on whether there were socioeconomic or ethnic differences between those who participated and those who did not. However, the majority of women in this study described already having a healthy diet, a physically active lifestyle and a supportive network of family and friends. It may be that women who felt less confident about these aspects declined to participate in the study.

## Conclusion

Prehabilitation may play an important role in the preparation of patients with ovarian cancer due to undergo major debulking surgery, however a standard multimodal model does not necessarily address the barriers and challenges to access that may be faced by a demographically diverse cohort. The individual components of prehabilitation and the way in which they are delivered are essential to consider in the context of each individual, in order to achieve effective personalised care.

## Electronic supplementary material

Below is the link to the electronic supplementary material.

Supplementary Material 1

## Data Availability

The datasets used and/or analysed during the current study are available from the corresponding author on reasonable request.

## Footnotes

1 A network of centres set up in the United Kingdom and Hong Kong to support anyone affected by cancer (15).

## Abbreviations

NHS: National Health Service
MILE: Marsden Integrated Lifestyle and Exercise
CNS: Clinical Nurse Specialist

## References

[1] Faithfull S, Turner L, Poole K, Joy M, Manders R, Weprin J, et al. Prehabilitation for adults diagnosed with cancer: A systematic review of long-term physical function, nutrition and patient-reported outcomes. Eur J Cancer Care (Engl). 2019;28(4):e13023. 30859650 10.1111/ecc.13023

[2] Prehabilitation guidance for healthcare professionals [Internet]. [cited 2021 Oct 8]. Available from: https://www.macmillan.org.uk/healthcare-professionals/news-and-resources/guides/principles-and-guidance-for-prehabilitation

[3] Giles C, Cummins S. Prehabilitation before cancer treatment. BMJ. 2019;366:l5120. 31413000 10.1136/bmj.l5120

[4] Tetsche MS, Dethlefsen C, Pedersen L, Sorensen HT, Norgaard M. The impact of comorbidity and stage on ovarian cancer mortality: A nationwide Danish cohort study. BMC Cancer. 2008;8(1):31. 18230177 10.1186/1471-2407-8-31 PMC2266760

[5] Ebell MH, Culp MB, Radke TJ. A systematic review of symptoms for the diagnosis of ovarian Cancer. Am J Prev Med. 2016;50(3):384–94. 26541098 10.1016/j.amepre.2015.09.023

[6] Saggu RK, Barlow P, Butler J, Ghaem-Maghami S, Hughes C, Lagergren P, et al. Considerations for multimodal prehabilitation in women with gynaecological cancers: a scoping review using realist principles. BMC Womens Health. 2022;22(1):300. 35854346 10.1186/s12905-022-01882-z PMC9294794

[7] Fotopoulou C, Planchamp F, Aytulu T, Chiva L, Cina A, Ergönül Ö et al. European Society of Gynaecological Oncology guidelines for the peri-operative management of advanced ovarian cancer patients undergoing debulking surgery. Int J Gynecol Cancer [Internet]. 2021 Sep 1 [cited 2021 Oct 8];31(9). Available from: https://ijgc.bmj.com/content/31/9/1199 10.1136/ijgc-2021-002951 34407962

[8] Macmillan Cancer Support. Cancer rehabilitation pathways guidance [Internet]. Macmillan Cancer Research; 2020 [cited 2021 Oct 6]. Available from: https://www.macmillan.org.uk/healthcare-professionals/news-and-resources/guides/cancer-rehabilitation-pathways-guidance

[9] Beck A, Vind Thaysen H, Hasselholt Soegaard C, Blaakaer J, Seibaek L. Prehabilitation in cancer care: patients’ ability to prepare for major abdominal surgery. Scand J Caring Sci. 2021;35(1):143–55. 32043644 10.1111/scs.12828

[10] Beck A, Vind Thaysen H, Hasselholt Soegaard C, Blaakaer J, Seibaek L. What matters to you? An investigation of patients’ perspectives on and acceptability of prehabilitation in major cancer surgery. Eur J Cancer Care (Engl). 2021;e13475. 10.1111/ecc.13475 34106493

[11] Beck A, Thaysen HV, Soegaard CH, Blaakaer J, Seibaek L. Investigating the experiences, thoughts, and feelings underlying and influencing prehabilitation among cancer patients: a qualitative perspective on the what, when, where, who, and why. Disabil Rehabil. 2020;1–8. 10.1080/09638288.2020.1762770 32400218

[12] van der Zanden V, van der Zaag-Loonen HJ, Paarlberg KM, Meijer WJ, Mourits MJE, van Munster BC. PREsurgery thoughts - thoughts on prehabilitation in oncologic gynecologic surgery, a qualitative template analysis in older adults and their healthcare professionals. Disabil Rehabil. 2021;1–11. 10.1080/09638288.2021.1952319 34283686

[13] Polen-De C, Langstraat C, Asiedu GB, Jatoi A, Kumar A. Advanced ovarian cancer patients identify opportunities for prehabilitation: A qualitative study. Gynecol Oncol Rep. 2021;36:100731. 33718562 10.1016/j.gore.2021.100731 PMC7910499

[14] Norell CH, Butler J, Farrell R, Altman A, Bentley J, Cabasag CJ, et al. Exploring international differences in ovarian cancer treatment: a comparison of clinical practice guidelines and patterns of care. Int J Gynecol Cancer. 2020;30(11):1748–56. 32784203 10.1136/ijgc-2020-001403 PMC7656152

[15] Fusch P, Ness L. Are We There Yet? Data Saturation in Qualitative Research. Walden Fac Staff Publ [Internet]. 2015;20(9). Available from: https://scholarworks.waldenu.edu/facpubs/455

[16] Braun V, Clarke V, Hayfield N. A starting point for your journey, not a Map’: Nikki hayfield in conversation with Virginia Braun and Victoria Clarke about thematic analysis. Qual Res Psychol. 2022;19(2):424–45.

[17] Tanay MAL, Armes J, Oakley C, Bryson L, Johnston R, Moss-Morris R et al. Co-designing a behavioural intervention for reducing the impact of chemotherapy-induced peripheral neuropathy symptoms: an evidence- and theory-driven approach. Eur J Cancer Care (Engl). 10.1111/ecc.13671 PMC9786800 35959639

[18] Skivington K, Matthews L, Simpson SA, Craig P, Baird J, Blazeby JM, et al. A new framework for developing and evaluating complex interventions: update of medical research Council guidance. BMJ. 2021;374:n2061. 34593508 10.1136/bmj.n2061 PMC8482308

[19] O’Cathain A, Croot L, Duncan E, Rousseau N, Sworn K, Turner KM, et al. Guidance on how to develop complex interventions to improve health and healthcare. BMJ Open. 2019;9(8):e029954. 31420394 10.1136/bmjopen-2019-029954 PMC6701588

[20] Wade-Mcbane K, King A, Urch C, Johansson L, Wells M. Is personalised prehabilitation feasible to implement for patients undergoing oncological treatment for lung cancer at a London teaching hospital? Protocol of a feasibility trial. BMJ Open. 2023;13(7):e072367. 37460263 10.1136/bmjopen-2023-072367 PMC10357652

[21] Understanding and Promoting Effective Engagement With Digital Behavior Change Interventions. Am J Prev Med. 2016;51(5):833–42. 10.1016/j.amepre.2016.06.015 27745683

[22] Patrick H, Williams GC. Self-determination theory: its application to health behavior and complementarity with motivational interviewing. Int J Behav Nutr Phys Act. 2012;9(1):18. 22385676 10.1186/1479-5868-9-18 PMC3323356

[23] Ohlsson-Nevo E, Alkebro I, Ahlgren J. Cancer patients’ interest in participating in cancer rehabilitation. Acta Oncol. 2019;58(12):1676–83. 31241428 10.1080/0284186X.2019.1633017

[24] Grimmett C, Bradbury K, Dalton SO, Fecher-Jones I, Hoedjes M, Varkonyi-Sepp J et al. The Role of Behavioral Science in Personalized Multimodal Prehabilitation in Cancer. Front Psychol [Internet]. 2021 [cited 2023 Jul 26];12. Available from: https://www.frontiersin.org/articles/10.3389/fpsyg.2021.634223 10.3389/fpsyg.2021.634223 PMC7921482 33664701

