# Supplementary material for: ‘I was eager to do anything I could to improve the situation’: a qualitative study of patients’ experiences and views of prehabilitation for ovarian cancer surgery
Source: BMC Womens Health. 2025 Mar 15;25:121. doi: 10.1186/s12905-025-03630-5 (PMC11909996; doi:10.1186/s12905-025-03630-5)
Supplement: Supplementary file 1 — Supplementary Material 1 [file 12905_2025_3630_MOESM1_ESM.docx]

Supplementary Material:

Topic guide for used in semi-structured interviews with advanced ovarian cancer patients

The following information will be relayed by the interviewer to the interviewee, prior to each interview:

*‘We have invited you here today to discuss your thoughts surrounding Prehabilitation. This is an intervention which focuses on preparing cancer patients for their treatment. Prehabilitation is often a combination of physical activity, nutritional advice and mental health and wellbeing. Some prehabilitation programmes may address alcohol, smoking and other factors affecting fitness leading up to treatment. Prehabilitation has been shown to improve how patients feel after their treatment, resulting in more effective rehabilitation and recovery. However, it is not well established for ovarian cancer patients who have to undergo abdominal surgery. In order to set up an effective model for prehabilitation, we would like to understand your views and experiences of preparation before and recovery after treatment, and whether prehabilitation is something you would consider useful’.*

**The pre-operative period:**

For us to make an effective Prehabilitation programme, it is important for us to know what women already do to prepare and what problems they face before surgery.

- Following your diagnosis of cancer, what did you do to prepare / are you preparing your body for surgery?
- Are you able to describe how you felt emotionally in the lead up to surgery and what you did to prepare yourself mentally, spiritually and emotionally?
- Could you talk me through the priorities that were uppermost in your mind in the lead up to your surgery?
- Are there ways in which you feel you could have been better prepared for your surgery?
- What else would you have liked in terms of support and preparation from the hospital (and community) team?

**Thoughts about Prehabilitation:**

As discussed above, Prehabilitation ensures that women are as fit and prepared as possible before their surgery, so that they can recover better and quicker.

- When you think about the period after surgery, what does recovery or recovering well mean/look like to you?
- (ICHT) How prepared are you/ would you be to make changes to your lifestyle before surgery? For example, make changes to your diet, activity or the way you manage your stresses/anxiety?
- (RMH) Tell me about your experience of Prehab so far?
- What do you think would be the most useful type of Prehabilitation?

**Feasibility of Prehabilitation:**

In order to make a successful programme, we need to understand how practical it would be/was for women to take part.

- Of all the things we have discussed so far, what do you think would appeal to women most?
- What would be easiest to incorporate into the weeks before surgery? (
- How practical would it be/was it to participate in a Prehabilitation programme in the weeks prior to your surgery?
- What do you think might prevent you from participating? Are there any barriers or challenges that you can identify?
- What would make you more likely to participate?

**Conceptualising Prehabilitation:**

When thinking about a Prehabilitation programme, it is important to consider how it is delivered so that we can help as many people in the most convenient way.

- What are your thoughts on visiting the hospital for additional prehab appointments in the lead up to your surgery compared with a virtual programme which you could access at home?
- How would you respond to written guidance only?

**Overview:**

- Having discussed prehabilitation what do you think are the most important issues for us to consider in developing a programme?
